# Low-iodine-dose computed tomography coupled with an artificial intelligence-based contrast-boosting technique in children: a retrospective study on comparison with conventional-iodine-dose computed tomography

**DOI:** 10.1007/s00247-024-05953-1

**Published:** 2024-06-05

**Authors:** Dong-Joo Shin, Young Hun Choi, Seul Bi Lee, Yeon Jin Cho, Seunghyun Lee, Jung-Eun Cheon

**Affiliations:** 1 Department of Radiology, Seoul National University Hospital, 101 Daehak-Ro, Jongno-Gu, Seoul, 03080 Republic of Korea; 2 Department of Radiology, Seoul National University College of Medicine, Jongno-Gu, Seoul, Republic of Korea; 3 Institute of Radiation Medicine, Seoul National University Medical Research Center, Jongno-Gu, Seoul, Republic of Korea

**Keywords:** Artificial intelligence, Computed tomography, Contrast enhancement, Image quality, Iodine, Pediatrics

## Abstract

**Background:**

Low-iodine-dose computed tomography (CT) protocols have emerged to mitigate the risks associated with contrast injection, often resulting in decreased image quality.

**Objective:**

To evaluate the image quality of low-iodine-dose CT combined with an artificial intelligence (AI)-based contrast-boosting technique in abdominal CT, compared to a standard-iodine-dose protocol in children.

**Materials and methods:**

This single-center retrospective study included 35 pediatric patients (mean age 9.2 years, range 1–17 years) who underwent sequential abdominal CT scans—one with a standard-iodine-dose protocol (standard-dose group, Iobitridol 350 mgI/mL) and another with a low-iodine-dose protocol (low-dose group, Iohexol 240 mgI/mL)—within a 4-month interval from January 2022 to July 2022. The low-iodine CT protocol was reconstructed using an AI-based contrast-boosting technique (contrast-boosted group). Quantitative and qualitative parameters were measured in the three groups. For qualitative parameters, interobserver agreement was assessed using the intraclass correlation coefficient, and mean values were employed for subsequent analyses. For quantitative analysis of the three groups, repeated measures one-way analysis of variance with post hoc pairwise analysis was used. For qualitative analysis, the Friedman test followed by post hoc pairwise analysis was used. Paired *t*-tests were employed to compare radiation dose and iodine uptake between the standard- and low-dose groups.

**Results:**

The standard-dose group exhibited higher attenuation, contrast-to-noise ratio (CNR), and signal-to-noise ratio (SNR) of organs and vessels compared to the low-dose group (all *P*-values < 0.05 except for liver SNR, *P* = 0.12). However, noise levels did not differ between the standard- and low-dose groups (*P* = 0.86). The contrast-boosted group had increased attenuation, CNR, and SNR of organs and vessels, and reduced noise compared with the low-dose group (all *P* < 0.05). The contrast-boosted group showed no differences in attenuation, CNR, and SNR of organs and vessels (all *P* > 0.05), and lower noise (*P* = 0.002), than the standard-dose group. In qualitative analysis, the contrast-boosted group did not differ regarding vessel enhancement and lesion conspicuity (*P* > 0.05) but had lower noise (*P* < 0.05) and higher organ enhancement and artifacts (all *P* < 0.05) than the standard-dose group. While iodine uptake was significantly reduced in low-iodine-dose CT (*P* < 0.001), there was no difference in radiation dose between standard- and low-iodine-dose CT (all *P* > 0.05).

**Conclusion:**

Low-iodine-dose abdominal CT, combined with an AI-based contrast-boosting technique exhibited comparable organ and vessel enhancement, as well as lesion conspicuity compared to standard-iodine-dose CT in children. Moreover, image noise decreased in the contrast-boosted group, albeit with an increase in artifacts.

**Graphical Abstract:**

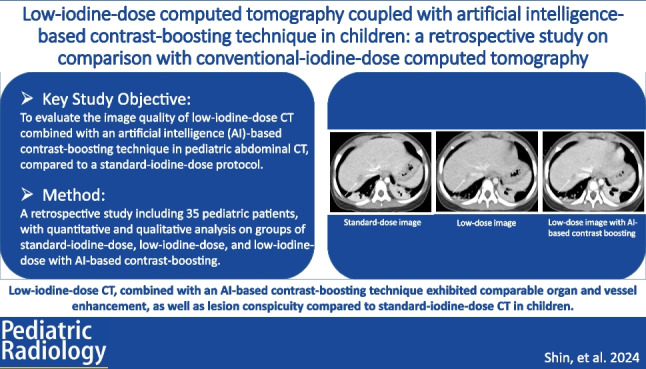

**Supplementary Information:**

The online version contains supplementary material available at 10.1007/s00247-024-05953-1.

## Introduction

Iodine contrast media in computed tomography (CT) is crucial for improving image visibility for accurate diagnosis. However, iodine contrast media can cause side effects—ranging from mild allergic reactions to severe complications, such as contrast-induced nephropathy, thyroid dysfunction, and anaphylactic reactions [1]. Notably, contrast-induced nephropathy presents a dose-dependent risk with contrast media usage [2], standing as the third most common cause (11%) of hospital-acquired renal failure [3]. In pediatric patients, the reported incidence of contrast-induced nephropathy stands at 10.3% following contrast-enhanced CT [4]. To mitigate the risk, the utilization of low- and isosmolar contrast media [5], along with the reduction of contrast dose [6], is recommended.

Numerous attempts have been made to reduce contrast dose while maintaining image quality. Utilizing a low kVp offers the advantage of reducing radiation dose while augmenting iodine contrast media attenuation, consequently decreasing the required contrast volume [7]. Thus, employing a low kVp allows for a “double low protocol” in pediatric patients, which reduces both radiation dose and contrast volume [8]. However, low kVp usage is often associated with increased image noise and artifacts. Technologies such as filtered back projection [9], iterative reconstruction [9], dual-energy computed tomography (DECT) [10–12], and deep learning reconstruction [13–15] have been instrumental in ameliorating image noise.

Recently, contrast-boosting using artificial intelligence (AI) techniques has been introduced to minimize contrast dose. AI-based contrast-boosting can be applied to any CT/magnetic resonance imaging (MRI) machine. Several studies have investigated the use of AI-based contrast-boosting in brain and cardiac MRI [16, 17], aortic CT angiography [18], and abdominal CT [19] in adult patients. These studies have reported a 10–50% reduction in contrast media. However, to our knowledge, no studies have investigated AI-based contrast-boosting in pediatric patients. Thus, this study aimed to evaluate the image quality of low-iodine-dose CT combined with an AI-based contrast-boosting technique in pediatric abdominal CT, compared with a standard-iodine-dose protocol.

## Materials and methods

This retrospective, single-center study was approved by our Institutional Review Board. The requirement for informed consent was waived.

### Patients

Based on earlier studies [8, 11], our institution adopted a low-iodine-dose protocol for pediatric abdominal CT scans in April 2022. In July 2022, we retrospectively reviewed the picture archiving and communication system (PACS) (Infinitt Healthcare, Seoul, South Korea) database for the period from January 2022 to July 2022 and searched for sequential abdominal CT scans—those with a standard iodine dose protocol (standard-dose group) and those with a low-iodine-dose protocol (low-dose group)—within a 4-month interval for the same patient. The standard-dose group were given Iobitridol 350 mgI/mL (Xenetix 350, Guerbet, Aulnay, France) with an iodine dose of 595 mgI/kg, while the low-dose group were given Iohexol 240 mgI/mL (Iobrix 240, Taejoon Pharmaceutical Co., Seoul, South Korea) with an iodine dose of 408 mgI/kg. Of the 46 potential participants, the following were excluded: (1) patients aged ≥ 18 years (*n* = 4), (2) patients whose body weight changed by more than 5% between the two scans (*n* = 5), and (3) images displaying severe motion or respiratory artifacts that impeded image interpretation (*n* = 2). Subsequently, a total of 35 pediatric patients were included in this study. 

### Computed tomography protocols

A 128-channel multidetector CT (MDCT) scanner (SOMATOM Definition Flash, Siemens AG, Forchheim, Germany) was used to obtain the CT images. In total, 1.7 mL/kg of contrast agent was administered over 50 s using a power injector (Envision CT, Medrad, Pittsburgh, PA). The average contrast agent volume was 55.2 ± 28.9 mL for the standard-dose protocol and 55.5 ± 28.5 mL for the low-dose protocol. The average injection rate was 1.1 ± 0.6 mL/s. The portal venous phase was obtained 70 s after contrast injection. DECT protocol was employed utilizing two X-ray tubes operating at different tube voltages—70 kV and 150 kV—with reference tube current–time products set at 370 mAs for 70 kVp and 93 mAs for 150 kV. Detailed CT scan parameters and contrast media usage for both standard- and low-dose CT are presented in Table 1.

**Table 1:** Computed tomography scan parameters and contrast media for each protocol

CT parameters	Standard-dose CT	Low-dose CT
Contrast medium	Iobitridol 350 mgI/mL	Iohexol 240 mgI/mL
Contrast media volume	1.7 ml/kg	1.7 ml/kg
Tube voltage (kVp)	70/Sn150	70/Sn150
Reference tube current (mAs)	370/93	370/93
Pitch (s)	0.6	0.6
Detector configuration	128 × 0.6 mm	128 × 0.6 mm
Gantry rotation time (s)	0.25	0.25
Slice thickness/interval (mm)	3/2	3/2
Scan timing	Portal phase (fixed delay of 70 s after injection)	
Reconstruction kernel	Body routine 36	Body routine 36

*CT* computed tomography

### Artificial intelligence-based contrast-boosting technique

For the AI-based contrast-boosting technique, ClariACE (ClariPi, Seoul, South Korea) was used. ClariACE has a two-stage U-net architecture to enhance contrast in low-contrast-dose CT. The details of ClariACE are described in Supplementary Material 1.

### Quantitative image analysis

Quantitative analysis was conducted by a third-year radiology resident (D-J.S.) who was blinded to clinical information. CT images were assessed in soft tissue window settings (width: 400 HU, level: 50 HU). The CT Hounsfield unit (HU) was measured on the axial image where the portal vein was observed at full width. Attenuations of liver parenchyma, portal vein, aorta, paraspinal muscle, and extracorporeal air were measured by manually drawing regions of interest (ROI) based on visual inspection. The ROIs were drawn at sites with homogeneous attenuation, and the size and shape of each ROI were applied equally to each organ. Regions with inhomogeneous attenuation due to vascular structures or beam-hardening artifacts were excluded from measurement. The average HU value of four separate ROIs in the right anterior, right posterior, left medial, and left lateral segments represented the attenuation of liver parenchyma. Similarly, the average HU value of two ROIs on both sides of the paraspinal muscles represented muscle attenuation. Image noise was determined as the average of two standard deviation (SD) values from both paraspinal muscles. The contrast-to-noise ratio (CNR) and the signal-to-noise ratio (SNR) were calculated as CNR = $({HU}_{organ}-{HU}_{muscle})$/${SD}_{muscle}$ and SNR = ${HU}_{organ}$/${SD}_{muscle}$, where ${HU}_{muscle}$ is the HU value of paraspinal muscle.

### Qualitative image analysis

Qualitative image analysis was conducted independently by D-J.S. and Y.H.C. (a pediatric radiologist with 17 years of experience), blinded to the clinical information. The readers evaluated organ enhancement, vessel enhancement, noise, artifact, overall image quality, and lesion conspicuity of the CT images, on a 5-point scoring scale. The grading scales for subjective image quality items are summarized in Table 2. Organ enhancement, vessel enhancement, overall image quality, and lesion conspicuity were scored higher in better image quality. Higher noise and artifact scores indicated less noise and minimal artifacts. Before assessing lesion conspicuity, all lesions were annotated on the PACS by a second pediatric radiologist (S.B.L. with 7 years of experience). The average qualitative scores from both readers were used for statistical analysis.

**Table 2:** Grading scale for subjective image quality

	Organ enhancement	Vessel enhancement	Noise	Artifact^a^	Overall image quality	Lesion conspicuity
5	Excellent	Excellent	Minimum or no noise	Minimum or no artifact	Excellent	Excellent
4	Above average	Above average	Less-than-average noise	Less-than-average artifact	Above average	Above average
3	Acceptable	Acceptable	Average noise	Average artifact	Acceptable	Acceptable
2	Suboptimal	Suboptimal	Above-average noise	Above-average artifact	Suboptimal	Suboptimal
1	Very poor	Very poor	Unacceptable noise	Unacceptable artifact	Very poor	Very poor

^a^Artifact was assessed as beam-hardening artifact, streak artifact, and parenchymal heterogeneity

### Radiation dose and iodine uptake

The CT dose index volume (CTDIvol, mGy) and dose-length products (DLP, mGy × cm) were recorded for all CT examinations based on CT dose reports. The effective dose (ED, mSv) was calculated as ED = DLP × K, where K is the conversion factor for the abdomen, which varies depending on kVP and age [20]. As the conversion factors for less than 80 kVp are unknown, a conversion factor of 80 kVp was applied instead. Linear interpolation was used to calculate the conversion factors for different ages and kVp values. The total iodine uptake for each patient was calculated as body weight (kg) × 1.7 mL/kg × iodine concentration (mgI/mL).

### Statistical analysis

Continuous variables were summarized as means and SD. Categorical variables were summarized as counts. For quantitative analysis, repeated measures one-way analysis of variance (ANOVA) with post hoc pairwise comparisons with Bonferroni correction was used to compare the three groups: standard-dose group, low-dose group, and low-dose group with AI-based contrast-boosting (contrast-boosted group). Qualitative scores were analyzed using the Friedman test, followed by post hoc Dunn’s pairwise comparisons. Interobserver agreement was assessed using the intraclass correlation coefficient (ICC): poor (< 0.20), fair (0.21–0.40), moderate (0.41–0.60), good (0.61–0.80), or excellent (0.81–1.00) [21]. The paired *t*-test was used to compare radiation dose and iodine uptake between the standard- and low-dose groups. All analyses were performed using MedCalc Software (version 22.009, MedCalc Software Ltd, Ostend, Belgium). Statistical significance was set at *P* < 0.05.

## Results

### Patient characteristics

A total of 35 patients (22 male) were included in the study. The mean age was 9.2 ± 4.9 years (range, 1–17 years). The mean body mass index (BMI) was 18.1 ± 3.6 kg/m^2^. The underlying diseases of the patients were as follows: lymphoma (*n* = 8), Ewing sarcoma (*n* = 7), neuroblastoma (*n* = 3), hepatoblastoma (*n* = 3), Wilms tumor (*n* = 3), rhabdomyosarcoma (*n* = 2), immature teratoma (*n* = 2), synovial sarcoma (*n* = 2), osteosarcoma (*n* = 1), ganglioneuroblastoma (*n* = 1), malignant peripheral nerve sheath tumor (*n* = 1), and leiomyosarcoma (*n* = 1). Ten lesions were used to evaluate lesion conspicuity, including liver mass (*n* = 3), lymph node enlargement (*n* = 2), adrenal mass (*n* = 1), spleen mass (*n* = 1), pleural mass (*n* = 1), retroperitoneal mass (*n* = 1), and bone mass (*n* = 1). The mean interval between the standard-dose and low-dose CTs was 80 ± 24 days [range, 23–125 days].

### Quantitative analysis

**Fig. 1:**
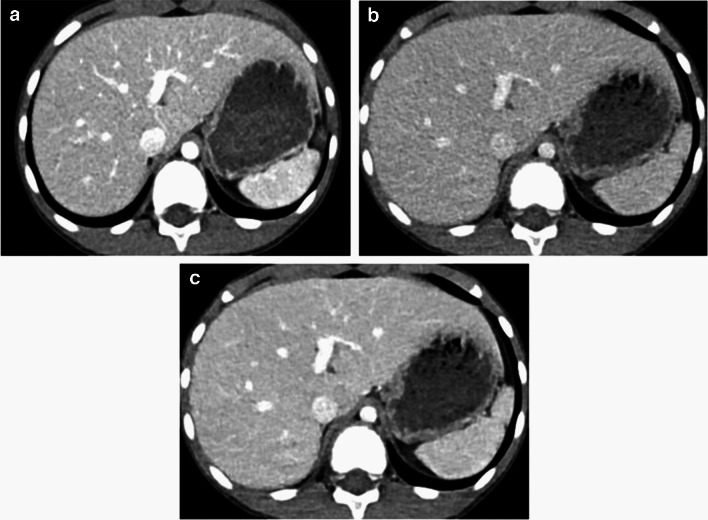
Axial contrast-enhanced computed tomography (CT) images of a 9-year-old boy with upper arm synovial sarcoma. Sequential abdominal CT was conducted within 2 months. **a** Standard-dose image shows excellent organ (liver, 167.6 ± 9.0 Hounsfield unit (HU)) and vessel (aorta, 327.0 ± 16.7 HU; portal vein, 324.8 ± 10 HU) enhancement with some grainy noise. **b** Low-dose image exhibits decreased organ (liver, 152.0 ± 12.1 HU) and vessel (aorta, 208.2 ± 12.6 HU; portal vein, 228.2 ± 14.9 HU) enhancement with persistent grainy noise. **c** Contrast-boosted image shows excellent organ (liver, 174.7 ± 9.5 HU) and vessel (aorta, 260.0 ± 11.9 HU; portal vein, 285.2 ± 12.1 HU) enhancement with reduced grainy noise. The qualitative organ enhancement scores for the standard-dose, low-dose, and contrast-boosted images were 4/4, 3/3, and 4/4, respectively (reader 1’s score/reader 2’s score). Vessel enhancement scores were 5/4, 3/3, and 4/4, whereas noise scores were 3/4, 3/3, and 4/4. Overall image quality scores were 4/4, 4/3, and 4/4

**Fig. 2:**
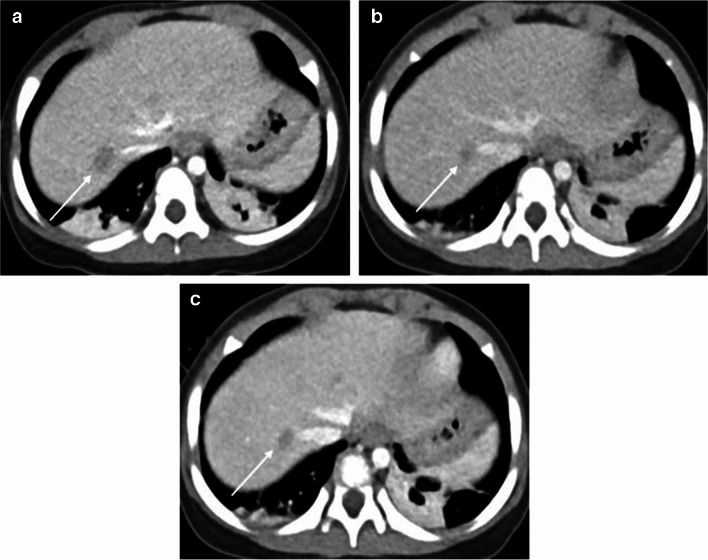
Axial contrast-enhanced computed tomography (CT) images of a 1-year-old girl with adrenal gland neuroblastoma and liver metastasis. Sequential abdominal CT was conducted within 3 months. **a** Standard-dose image shows a 1.3 cm relatively well-defined low attenuated metastatic nodule in liver segment 7/8 (*arrow*). **b** Low-dose image shows a 1.1-cm ill-defined low attenuated metastatic nodule in liver segment 7/8 (*arrow*). **c** In the contrast-boosted image, the nodule looks more well-defined (*arrow*) and the lesion conspicuity is improved compared to the low-dose image. The qualitative lesion conspicuity scores of the standard-dose, low-dose, and contrast-boosted images were 4/4, 3/3, and 4/4, respectively (reader 1’s score/reader 2’s score)

**Fig. 3:**
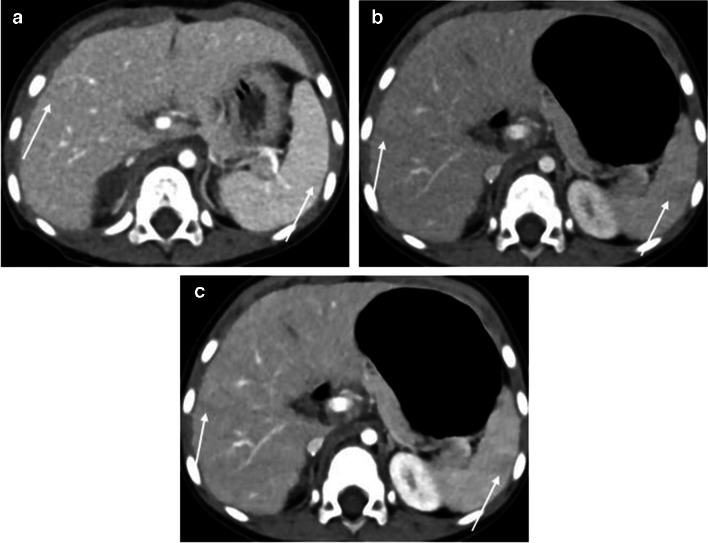
Axial contrast-enhanced computed tomography (CT) images of a 2-year-old girl with immature teratoma. Sequential abdominal CT was conducted within 4 months. **a** Standard-dose image shows a minimal streak artifact underneath both ribs (*arrows*). **b** Low-dose image shows a mild streak artifact underneath both ribs (*arrows*). **c** In the contrast-boosted image, the streak artifact is more prominent in the more enhanced liver and spleen (*arrows*). The qualitative artifact scores of the standard-dose, low-dose, and contrast-boosted images were 5/4, 4/4, and 3/3, respectively (reader 1’s score/reader 2’s score)

The results of the quantitative analysis of the three groups are summarized in Table 3. The standard-dose group had higher attenuation, CNR, and SNR than the low-dose group (all *P*-values < 0.05 except for liver SNR, *P* = 0.12). No significant difference in noise was observed between the standard- and low-dose groups (*P* = 0.86). In the contrast-boosted group, attenuation, CNR, and SNR of organs and vessels were significantly increased (all *P* < 0.001), while noise decreased (*P* = 0.04) than the low-dose group. The contrast-boosted group showed no significant differences in attenuation, CNR, and SNR of organs and vessels (all *P* > 0.05) and lower noise (*P* = 0.002) compared to the standard-dose group (Figs. 1, 2, and 3).

**Table 3:** Results of quantitative analysis

		350 mgI/mL	240 mgI/mL	240 mgI/mL + contrast boosting	*P*-value^a^	Subgroup comparison^a^
Hounsfield units	Liver	146.8 ± 19.3	126.7 ± 13.5	146.7 ± 19.7	< 0.001	< 0.001^b^ < 0.001^c^ 1.00^d^
	Aorta	241.2 ± 42.7	185.7 ± 30.9	234.8 ± 42.7	< 0.001	< 0.001^b^ < 0.001^c^ 1.00^d^
	Portal vein	252.2 ± 41/7	193.0 ± 28.7	242.3 ± 40.3	< 0.001	< 0.001^b^ < 0.001^c^ 0.21^d^
	Muscle	79.9 ± 6.7	73.9 ± 5.4	79.2 ± 7.0	< 0.001	< 0.001^b^ < 0.001^c^ 1.00^d^
Noise		9.4 ± 2.4	8.9 ± 2.2	8.3 ± 1.9	< 0.001	0.86^b^ 0.04^c^ 0.002^d^
Contrast-to-noise ratio	Liver	7.6 ± 2.8	6.2 ± 2.0	8.4 ± 2.7	< 0.001	0.004^b^ < 0.001^c^ 0.32^d^
	Aorta	18.2 ± 6.3	13.2 ± 4.9	19.5 ± 5.9	< 0.001	< 0.001^b^ < 0.001^c^ 0.73^d^
	Portal vein	19.2 ± 5.4	13.9 ± 3.9	20.3 ± 5.0	< 0.001	< 0.001^b^ < 0.001^c^ 0.70^d^
Signal-to-noise ratio	Liver	16.8 ± 5.1	15.0 ± 3.9	18.5 ± 4.3	< 0.001	0.12^b^ < 0.001^c^ 0.27^d^
	Aorta	27.4 ± 8.2	22.0 ± 6.6	29.5 ± 7.3	< 0.001	0.001^b^ < 0.001^c^ 0.45^d^
	Portal vein	28.4 ± 7.3	22.7 ± 5.6	30.3 ± 6.4	< 0.001	< 0.001^b^ < 0.001^c^ 0.46^d^

^a^Repeated measures one-way analysis of variance with post hoc pairwise comparisons with Bonferroni correction

^b^350 mgI/mL vs. 240 mgI/mL

^c^240 mgI/mL vs. 240 mgI/mL with contrast boosting

^d^350 mgI/mL vs. 240 mgI/mL with contrast boosting

### Qualitative analysis

The results of the qualitative analysis of the three groups are summarized in Table 4. Interobserver agreement was assessed using ICC. ICC of qualitative scores of the two readers were 0.37–0.83, indicating a fair to excellent level of concordance (Table 5). The standard-dose group showed higher organ and vessel enhancement, overall image quality, lesion conspicuity, and fewer artifacts than the low-dose group (all *P* < 0.05). No significant difference in noise was observed between the standard- and low-dose groups (*P* < 0.05). In the contrast-boosted group, organ and vessel enhancement, overall image quality, lesion conspicuity, and artifact were greater than those in the low-dose group (all *P* < 0.05), while noise decreased in the contrast-boosted group (*P* < 0.05). The contrast-boosted group showed no difference in vessel enhancement and lesion conspicuity (*P* > 0.05), lower noise (*P* < 0.05), and higher organ enhancement and artifact (all *P* < 0.05) than the standard-dose group (Figs. 1, 2, and 3).

**Table 4:** Results of qualitative analysis

	350 mgI/mL	240 mgI/mL	240 mgI/mL + contrast boosting	*P*-value^a^	Subgroup comparison^a^
Organ enhancement	4.4 ± 0.4	3.6 ± 0.3	4.6 ± 0.3	< 0.001	< 0.05^b^ < 0.05^c^ < 0.05^d^
Vessel enhancement	4.7 ± 0.3	3.6 ± 0.7	4.6 ± 0.4	< 0.001	< 0.05^b^ < 0.05^c^ > 0.05^d^
Noise	3.9 ± 0.4	3.9 ± 0.5	4.1 ± 0.4	< 0.001	> 0.05^b^ < 0.05^c^ < 0.05^d^
Artifact	4.5 ± 0.3	4.1 ± 0.4	3.6 ± 0.5	< 0.001	< 0.05^b^ < 0.05^c^ < 0.05^d^
Overall image quality	4.4 ± 0.3	3.7 ± 0.5	4.2 ± 0.3	< 0.001	< 0.05^b^ < 0.05^c^ < 0.05^d^
Lesion conspicuity	4.4 ± 0.5	3.3 ± 0.6	4.3 ± 0.4	< 0.001	< 0.05^b^ < 0.05^c^ > 0.05^d^

^a^Friedman test, followed by post-hoc pairwise comparison

^b^350 mgI/mL vs. 240 mgI/mL

^c^240 mgI/mL vs. 240 mgI/mL with contrast boosting

^d^350 mgI/mL vs. 240 mgI/mL with contrast boosting

**Table 5:** Interobserver agreement for qualitative assessment

	Intraclass correlation coefficient (average measures)	95% confidence interval
Organ enhancement	0.65	0.49 ~ 0.76
Vessel enhancement	0.83	0.75 ~ 0.88
Noise	0.37	0.08 ~ 0.57
Artifact	0.50	0.27 ~ 0.66
Overall image quality	0.66	0.50 ~ 0.77
Lesion conspicuity	0.61	0.19 ~ 0.82

### Radiation dose and iodine uptake

The results of radiation dose and iodine uptake are presented in Table 6. A significant reduction in iodine uptake (31.1%) was achieved by using low-iodine-dose CT (*P* < 0.001). There was no significant difference in radiation dose between standard- and low-iodine-dose CT (all *P* > 0.05).

**Table 6:** Radiation dose and iodine reduction effect

	350 mgI/mL	240 mgI/mL	*P*-value (paired *t*-test)
CTDI_vol_ (mGy)	2.0 ± 1.0	2.1 ± 1.3	0.82
DLP (mGy*cm)	94.4 ± 67.1	97.6 ± 77.7	0.56
ED (mSv)	2.36 ± 1.1	2.39 ± 1.3	0.82
Iodine uptake (mg/I)	19,320 ± 10,113	13,317 ± 6,846	< 0.001

*CTDI*_vol_ computed tomography dose index volume*, DLP* dose-length product*, ED* effective dose

## Discussion

This study revealed the possibility of maintaining image quality while reducing contrast media dosage in pediatric abdominal CT scans by applying an AI-based contrast-boosting technique to low-iodine-dose CT. The low-dose group alone showed lower image quality compared to the standard-dose group. However, with the combination of ClariACE, both quantitative and qualitative measures of image quality demonstrated no significant difference compared to the standard-iodine-dose group. There were comparable results regarding organ and vessel enhancement as well as lesion conspicuity between the standard-dose group and the contrast-boosted group. Additionally, the contrast-boosted group exhibited lower noise levels than the standard-dose group. Consequently, a 31.1% reduction in iodine load was achieved by applying the AI-based contrast-boosting technique.

A high dose of contrast media increases the risk of contrast-induced nephropathy in pediatric patients [22]. Thus, using low-concentration contrast media may reduce the risk of contrast-induced nephropathy, even without changing the contrast volume and injection rate. In addition, high osmolality and viscosity are related to a higher risk of contrast media extravasation and acute tissue injury [23, 24]. Patient discomfort during contrast injection, such as pain or heat sensation, is also related to the higher osmolality of contrast media [25]. The contrast media used in the low-dose group (Iohexol 240 mgI/mL) has lower osmolality (520 vs. 915 mOsm/kg water) and viscosity (3.4 mPa·s vs. 10 mPa·s at 37 °C) compared to that in the standard-dose group (Iobitridol 350 mgI/mL). Thus, using Iohexol 240 not only reduces the risk of dose-dependent adverse reactions but also may help acquire better image quality with reduced motion artifact in children by alleviating patient discomfort during contrast injection.

However, reducing iodine concentration diminishes organ and vessel enhancement, thereby compromising image visibility. Various technologies have been introduced to overcome image quality concerns stemming from contrast dose reduction. First, employing a low tube voltage was explored, which increases iodine contrast enhancement but concurrently elevates image noise. Iterative reconstruction techniques were used to compensate for the increased image noise. However, denoising via iterative reconstruction has limitations as higher weights of iterative reconstruction algorithms may diminish spatial resolution [26].

Second, DECT can be used to reconstruct virtual monoenergetic images at various keV levels by acquiring two image sets at two different peak energy levels. The attenuation of iodine contrast can be amplified by the reconstruction of low-keV images. However, it is not widely adopted due to the necessity for specialized CT equipment supporting dual-energy CT protocols. Cross-scattered radiation is another issue that can produce artifacts and decrease the CNR of images [27].

Third, contrast augmentation using AI techniques has emerged. It is accurate, computationally efficient, and can be applied to any CT or MRI machine. As demonstrated in our study, AI-based contrast-boosting technique can reduce contrast dose as well as image noise. Previous studies on AI-based contrast-boosting have reported its superior diagnostic values in CT/MR angiography [18, 28] and hypervascular lesions such as hepatocellular carcinoma (HCC) [19]. In our study, we observed that not only hypervascular lesions but also hypovascular lesions can be detected more easily in images with AI-based contrast-boosting because the overall background organ enhancement is increased.

However, while AI-based contrast-boosting increases organ and vessel enhancement, it also increases artifacts. This may be due to the ClariACE technique increasing the attenuation of high-attenuated artifacts such as beam-hardening artifacts, as well as normal structures. Additionally, low attenuated streak artifacts can be observed more prominently in organs with increased parenchymal enhancement. Thus, baseline images should be more homogeneous and lack artifacts in order to obtain better contrast-boosted images. Unfortunately, the dose setting of pediatric CT in our study was generally low (the CTDI_vol_ was 2.0–2.1 and DLP was approximately 94–97 mGy × cm), therefore the baseline image quality was not perfect. If the radiation dose had been set at a higher level, the artifacts would have been ameliorated in the contrast-boosted images.

This study has several limitations. First, only 35 patients were included, of whom only 10 had lesions to evaluate for conspicuity. The small sample size is attributed to the implementation of a low-iodine-dose CT protocol in pediatric patients at our institute since April 2022, resulting in few patients undergoing standard-dose CT thereafter. Furthermore, the number of patients meeting the criteria for sequential abdominal CT with varying doses at short intervals was also limited. However, the majority of significant results in our study yielded extremely low *P*-values of < 0.001, bolstering the credibility of our findings. Second, the ICC for qualitative analysis did not reach a sufficiently high level to evaluate noise and artifacts. This limitation may stem from the inherent subjectivity of assessment between the two readers. Nevertheless, the consistent results observed in quantitative analysis regarding noise validate the overall agreement. Finally, there existed a brief yet noticeable time gap between the two sequential CT scans, during which some patients underwent treatment. Consequently, the patient and lesion statuses were not identical between the two scans, potentially compromising the accuracy of comparisons.

## Conclusion

In conclusion, our findings suggest that low-iodine-dose abdominal CT, coupled with an AI-based contrast-boosting technique, yields comparable organ and vessel enhancement, as well as lesion conspicuity compared to standard-iodine-dose CT in pediatric patients. Moreover, the AI-based contrast-boosted group exhibited decreased image noise, albeit with an increase in artifacts. Approximately 30% of iodine uptake reduction was achieved without impairing image quality or lesion conspicuity.

### Supplementary Information

Below is the link to the electronic supplementary material.

Supplementary file1 (DOCX 77 KB)

## Data Availability

The datasets generated during and/or analyzed during the current study are available from the corresponding author on reasonable request.
