# Correction to: Low‑iodine‑dose computed tomography coupled with an artificial intelligence‑based contrast‑boosting technique in children: a retrospective study on comparison with conventional‑iodine‑dose computed tomography

**DOI:** 10.1007/s00247-024-05996-4

**Published:** 2024-07-04

**Authors:** Dong‑Joo Shin, Young Hun Choi, Seul Bi Lee, Yeon Jin Cho, Seunghyun Lee, Jung‑Eun Cheon

**Affiliations:** 1 Department of Radiology, Seoul National University Hospital, 101 Daehak‑Ro, Jongno‑Gu, Seoul, 03080 Republic of Korea; 2 Department of Radiology, Seoul National University College of Medicine, Jongno‑Gu, Seoul, Republic of Korea; 3 Institute of Radiation Medicine, Seoul National, University Medical Research Center, Jongno‑Gu, Seoul, Republic of Korea

**Correction to: Pediatric Radiology (2024)**

10.1007/s00247-024-05953-1

The authors state that in their original submission and therefore in the online published version, they

1. Named the contrast agent as “Iobx 240, Taejoon Pharmaceutical Co., Seoul, Korea”.

This was an error and should read,

“Iobrix 240, Taejoon Pharmaceutical Co., Seoul, South Korea”.

2. Declared,

“**Conflicts of interest** None”

This was an error and should read,

“**Conflicts of interest** A condition of the funders of this project (Taejoon Pharmaceutic Co., Seoul, South Korea) was that the research team should use the company’s own contrast agent (Iobrix 240) in the conduct of the study.”

The original article has been corrected.

