# Supplementary material for: Low-iodine-dose computed tomography coupled with an artificial intelligence-based contrast-boosting technique in children: a retrospective study on comparison with conventional-iodine-dose computed tomography
Source: Pediatr Radiol. 2024 Jun 5;54(8):1315–24. doi: 10.1007/s00247-024-05953-1 (PMC11254996; doi:10.1007/s00247-024-05953-1)
Supplement: Supplementary file 1 — Supplementary file1 (DOCX 77 KB) [file 247_2024_5953_MOESM1_ESM.docx]

Supplementary Material 1

The Deep Learning-based Iodine Contrast Augmenting Algorithm (DLICA) was designed to enhance contrast in low-contrast-dose contrast-enhanced computed tomography (CE CT) using a two-stage U-net architecture and a training dataset derived from dual-energy CT (DECT). The training process involved generating synthetic low-contrast-dose CE CT images with varying degrees by combining virtual non-contrast (VNC) and weight-adjusted iodine component images from DECT, with weight ranges from 0.5 to 1.5.

The first stage of the U-net is a pre-trained model network for image denoising, previously developed and validated. The second stage is trained to take a synthetic low-contrast CE CT image as input and predict the weight-adjusted iodine component image. DLICA then produces the contrast-augmented image by multiplying the predicted iodine component image with a user-defined boosting strength and adding it back to the input image.

DLICA offers users the flexibility to adjust the boosting strength for contrast enhancement and provides an option for denoising strength in the first stage of the process. This algorithm represents a sophisticated approach to improving contrast in low-dose CT imaging through deep learning techniques and dual-energy CT-derived training data.


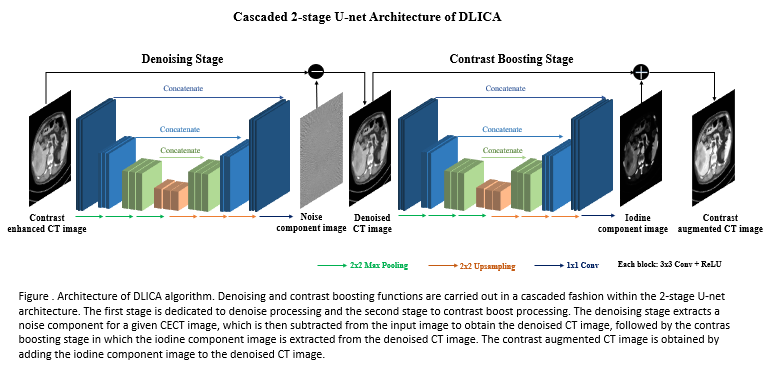
 Figure 1. The DLICA algorithm employs a two-stage U-net architecture for denoising and contrast augmentation in a cascaded fashion. The first stage focuses on denoising, extracting a noise component from the given contrast-enhanced CT (CECT) image, and subtracting it from the input to obtain a denoised CT image. In the second stage, dedicated to contrast augmentation, the iodine component image is extracted from the denoised CT image, and the final contrast-augmented CT image is generated by adding this iodine component image back to the denoised CT image. This sequential approach within the U-net framework aims to enhance contrast in low-contrast-dose CECT images by first reducing noise and then selectively augmenting contrast components.

Reference

Lee, T. H. et al. (2023) Deep learning‑based iodine contrast‑augmenting algorithm for low‑contrast‑dose liver CT to assess hypovascular hepatic metastasis. Abdo Radiol 48:3430–3440
